# Blood biomarkers for the prediction of idiopathic taste dysfunction: a case-control study

**DOI:** 10.1007/s00405-026-10455-x

**Published:** 2026-07-19

**Authors:** Mariano Mastinu, Stephan Habiger, Siegfried Heckmann, Thomas Hummel

**Affiliations:** 1 Department of Otorhinolaryngology, Smell & Taste Clinic, Technische Universität Dresden, Dresden, Germany; 2 Department of Biomedical Sciences, University of Cagliari, Monserrato, 09042 Italy; 3 Dental Practice, Hof, Germany; 4 Department of Dental Prosthetics and Materials Science, University Clinic of the Saarland, Homburg, Germany

**Keywords:** Dysgeusia, Idiopathic taste loss, Inflammatory biomarkers, Microelements, Salivary potassium

## Abstract

**Purpose:**

Taste dysfunction affects quality of life, yet its biological development remains poorly understood.

**Methods:**

We investigated clinical and biochemical differences between 50 patients with idiopathic dysgeusia and 102 healthy controls (110 women, 42 men; mean age 46.4 ± 1.4 years). Each participants underwent a clinical anamnesis, a taste test, and a blood analysis.

**Results:**

Patients were older (61 vs. 39 years; *p* < 0.001) and more often female (86% vs. 66%; *p* = 0.009). Patients had lower Taste Strips scores, and higher scores on depression (Beck Depression Index) and mood-related questionnaires (Zerssen Mood Scale; both p < 0.001). Blood analyses revealed that patients had higher levels of complement C4 (*p* = 0.011) and calcium (*p* = 0.019 ANCOVA, age-controlled). A logistic regression model including age, gender, C4, Ca, K, and transferrin predicted taste dysfunction with high accuracy (Nagelkerke R²=0.74; 91.4% correctly classified). Increased age, higher C4 and female sex were associated with higher odds of taste dysfunction, while higher potassium, transferrin levels, and salivary calcium were protective (*p* ≤ 0.030).

**Conclusion:**

These findings suggest that taste dysfunction is accompanied by systemic inflammatory conditions and lower levels of electrolytes in serum may contribute to development of taste diseases. Overall, the results suggest that idiopathic dysgeusia appears to be related to metabolic imbalance.

## Introduction

Taste plays a vital role in determining food safety and influencing daily eating behaviors [1]. Disturbances in taste perception can significantly impact nutritional intake, overall health, and quality of life, potentially leading to decreased appetite, weight loss, or, conversely, increased intake of unhealthy foods to compensate for the diminished taste sensation [2].

Viral infections, head trauma, and surgeries are the most common causes of taste dysfunction. Additionally, gustatory function is subject to a physiological decline in the elderly: age plays a critical role. However, there are numerous cases where the cause of dysgeusia is not clear [3]. Even a thorough medical history is not sufficient to explain the reason for the change in perception. Therefore, idiopathic taste disorders present a unique challenge in diagnosis let alone management [4].

Inflammation has been shown to be a common factor in many of the conditions and diseases associated with taste disorders [5]. Autoimmune diseases, such as Sjögren’s syndrome and systemic lupus erythematosus, are known to affect taste function as well [6, 7]. Although not completely understood, specific Toll-like Receptor (TLR) genes are specifically expressed in taste bud cells, enhancing the inflammatory response in gustatory regions [8], suggesting a potential path for future investigation in these patients. Nowadays, the best studied example is the taste disorder following SARS-CoV-2 infections. The interaction of COVID-19’s spike protein with the ACE receptors triggers several molecular processes in the infected cells that result in the production of IL-6 and other proinflammatory cytokines including interferons [9–11]. Recent evidence suggested that patients reporting loss of chemosensory functions during the time of infection were associated with higher mucosal antibody responses, pointing toward a role of local immune mechanisms in the persistence of these symptoms. These inflammatory processes can trigger apoptotic cell death and therefore may cause abnormal cell turnover in taste buds [7]. However, only few studies investigated the correlation between inflammatory parameters and chemosensory dysfunction [11, 12]. In both in vivo and animal model, acute inflammatory signals rapidly and directly alter peripheral taste bud sodium (ENaC) signaling, providing a mechanistic basis for dysgeusia during systemic illness [13]. However, Schalk and colleagues found no correlation between C-reactive protein or leukocyte counts with taste perception. Hence, other inflammatory pathways are likely to also contribute to taste disturbances.

On the other hand, saliva plays a key role in taste perception. Salivary proteins, metallic elements, and metalloproteinases interact with chemical compounds before reaching taste buds. Variations in chemical composition can contribute to an imbalanced taste perception [14]. Among others, zinc is an essential trace element playing a significant role in taste. Zinc is particularly important for metalloproteins as carbonic anhydrase IV (CAIV), which acts as trophic factor for the development of taste papillae. Some studies have already reported the importance of zinc for taste function, and that patients with taste impairment might exhibit malabsorption of zinc from their diet [15]. Based on double-blind randomized clinical trials, zinc therapy was shown to be efficient [16–18]. However, literature reports inconsistent findings regarding the efficacy on zinc supplementation in improving taste disorders [19, 20]. Similarly, iron can impair taste function: lower serum iron was associated with dysgeusia [21].

Although there are growing numbers of studies on possible causes of idiopathic dysgeusia, today still little is known about the possible inflammatory and metabolic causes of idiopathic dysgeusia. Because of this gap, we aimed to examine blood biochemistry and microelements in saliva in patients with taste dysfunction and compare them to healthy controls, in order to assess which biomarkers might work as potential predictors for idiopathic taste disorders.

## Materials and methods

### Ethical statement

The study was conducted in accordance with the Declaration of Helsinki and was approved by the Ethics Committee of the Medical Faculty at the University of Erlangen-Nürnberg (Nr. 2266). After receiving comprehensive information about the aims and possible risks of their participation in the study, all participants provided their written consent.

### Procedure

Patients were recruited at the Outpatient Clinic for Dental Prosthetics at the Erlangen Dental, Erlangen (Germany). They were excluded when taking medication known to cause taste disorders due to possible general illnesses. All patients underwent a detailed interview based on dysgeusia and medical history questionnaires [22]. Patients with dysgeusia associated with ENT surgery, viral infection, neurological or metabolic diseases, or occurring alongside burning mouth syndrome were excluded from the study. Only patients with dysgeusia in absence of identifiable causes based on clinical history and ENT examination were included. Their mood was assessed using Beck’s depression index [23] and the Zerssen mood scale [24]. Evaluation of their chemosensory function was conducted with validated psychophysical tests that are used in clinical practice. Finally, blood and saliva samples were taken for further analysis. Participants provided unstimulated saliva samples in the morning and were instructed not to eat or drink for at least 1 h prior to testing.

### Questionnaires

Depressive symptoms were assessed using the Beck’s Depression Inventory (BDI), a self-reported questionnaire that comprises 21 items rated on a 4-point scale (0–3) according to symptom severity, such as sadness, changes in appetite, or sexual behavior, over the preceding two weeks. Scores ranging from 0 to 13 indicate no or minimal depression, 14 to 19 correspond to mild depression, 20 to 28 to moderate depression, and scores between 29 and 63 reflect severe depressive symptoms [23, 25]. Mood and subjective well-being were assessed using the Zerssen mood scale (BEF) [24], a standardized questionnaire developed to evaluate transient changes in psychological state. The instrument consists of 16 adjective pairs representing different dimensions of affective state. Total scores ranged from 0 to 56, with higher values indicating poorer mood and lower scores reflecting well-being [24].

### Chemosensory tests

Gustatory functions were evaluated by using a standardized taste test kit, the “Taste strips” in a lateralized fashion [26]. The test comprises of sixteen taste-impregnated paper filter strips with an increasing concentration of the 4 basic taste (sweet, sour, bitter, salty). The strips were presented in a semi-randomized order, with the lower concentrated ones presented at first, alternatively in the left or in the right side of the tongue for assessing lateralized taste perception. Trained examiners conducted the study by placing the strip on the left or right side of the anterior part of the extended tongue, and participants were instructed to identify the taste quality among four options before bringing the tongue back in the oral cavity. After each strip, participants rinsed their mouth with water. The interstimulus interval was approximately 30 s. The total score was based on the sum of correct identification for both sides, ranging from 0 to 32. Participants were required to refrain from eating or drinking anything (except non-carbonated/tap water) one hour prior to the beginning of the experimental procedure.

Olfactory function was assessed with the Identification subset of the Sniffin’ Sticks test [27] as a standard screening method in clinical practice. Briefly, sixteen felt-tip pens containing an odor were presented orthonasally to the participant. They had to identify the odor using four different descriptors per pen. A correct identification corresponds to 1 point, and total score if 16 is given by the sum of correct responses.

### Blood /saliva analyses

Each participant underwent a blood analysis, which included the following parameters known to be associated with taste dysfunction: Serum iron, zinc levels and iron metabolism (ferritin, transferrin), serum electrolytes, hematocrit, complete blood count, liver values (transaminases), kidney function (creatinine), rheumatoid factors, complement C3/C4 and ANA titers. If values suggested a pathological event in the sense of a general disease, further diagnostics were carried out on a case-by-case basis. The selected blood parameters were chosen to provide a comprehensive assessment of systemic conditions known to influence taste function.

All patients provided an unstimulated saliva sample. The sample volume was 2 ml for all participants. The following parameters were determined: zinc, sodium, potassium, calcium, and chloride levels.

### Statistical analysis

Descriptive statistics were used to summarize socio-demographic characteristics of the study population. Continuous variables were expressed as mean ± standard error (SE). Categorical variables were reported as frequencies and percentages and compared using chi-square (χ^2^) test. Normal distribution of data was assessed using skewness ratio [28]. Age difference between groups was assessed with one-way ANOVA. Given the difference, the following comparisons were run using age as covariate for one-way ANCOVA. To identify biomarkers predictive of idiopathic dysgeusia, a binary logistic regression was performed, and Odds Ratios (ORs) and corresponding 95% confidence intervals (CIs) were calculated. Collinearity was assessed using variance inflation factors (VIF). Additionally, model discrimination was assessed using Receiver Operating Characteristic (ROC) analysis and the area under the curve (AUC). Internal validation was performed using bootstrap resampling (1000 iterations) to assess the stability of model coefficients, and 95% confidence intervals (95% CI) are reported. The software package SPSS 29.0 (Statistical Packages for Social Sciences Inc., Chicago, Ill., USA) was employed for statistical evaluation. The results are presented as the means ± SE. For visualization, R statistical software (R Development Core Team 2008, version 2023.06.0 for Windows), with the library “ggplot2” was used.

## Results

A total of 152 participants were included (110 women, 42 men, mean age 46.4 ± 1.4 years). Participants were divided into patients with idiopathic taste dysfunction (*n* = 50) and healthy controls (*n* = 102). One way ANOVA revealed that patients with taste dysfunction were older than healthy controls (61.0 vs. 39.2; *p* < 0.001). Table 1 shows a summary of demographic and clinical data.

**Table 1:** Anthropological characteristics and chemosensory tests comparisons

	Control group (*n* = 102)		Patient group (*n* = 50)		
	mean	SE	mean	SE	*p* value
Age [years]	39.2	1.3	61	1.9	< 0.001^a^
*Gender*					
Women	67	65.7%	43	86.0%	
Men	35	34.3%	7	14.0%	0.009^b^
Duration of disease [months]			43.1	7.9	
*Type of Dysgeusia*					
sweet			1	2%	
sour			13	26%	
bitter			23	46%	
salty			13	26%	
*Sniffin Sticks*	14.0	0.2	12.3	0.3	< 0.001
*Taste Strip Test*					
*Right*	12.9	0.3	10.2	0.5	< 0.001
Sweet	3.4	0.1	3.2	0.1	–
Sour	3.1	0.1	2.1	0.2	< 0.001
Bitter	3.2	0.1	2.4	0.2	< 0.001
Salty	3.2	0.1	2.5	0.1	< 0.001
*Left*	13.1	0.3	10.1	0.4	< 0.001
Sweet	3.5	0.1	3.0	0.1	0.01
Sour	3.2	0.1	2.2	0.1	< 0.001
Bitter	3.2	0.1	2.5	0.1	< 0.001
Salty	3.3	0.1	2.4	0.1	< 0.001
*Questionnaires*					
BDI	2.1	0.6	9.6	1.0	< 0.001
BEF	3.6	0.9	15.8	1.3	< 0.001

*p*-values after one-way-ANCOVA controlled by age. ^a^ *p*-value after one-way ANOVA; ^b^ *p*-value after chi-square (χ^2^) test

Compared to patients, healthy controls had a higher percentage of males (χ^2^ = 6.92, *p* = 0.009), and they scored higher for the Taste strip test and identification of all single taste qualities (*p* < 0.001), except for sweet on the right side which was not significant. Patients obtained a significant lower score in smell identification test (*p* < 0.001), and in both questionnaires (*p* < 0.001).

Table 2 shows serum microelements, cell counts and protein profile in controls and patients. One-way ANCOVA controlled by age showed that patients had higher values of complement C4 and Calcium (Ca) and a tendency for lower concentration of Potassium (K) and Blood Sedimentation Rate (BSR), which missed the significant cut-off.

**Table 2:** Comparisons of biomarkers between groups

	Control group (*n* = 102)		Patient group (*n* = 50)			
	mean	±SE	mean	±SE	*p* value	Range
*Blood routine*						
Erythrocytes	4.8	0.1	4.8	0.1	–	4.5–5.9 millions/µL
Leukocytes	6.9	0.2	6.5	0.3	–	3.8–10.8 × 103 /µl
Mean Corpuscular Volume	90.5	0.7	88.5	1.0	–	80–96 fl.
Major Histocompatibility Complex	33.3	2.6	29.1	4.0	–	26–32 pg/mL
Mean Corpuscular Hemoglobin Concentration	32.9	0.1	32.6	0.2	–	32–36%
Thrombocytes	262.6	0.1	264.2	10.3	–	150–450 Gpt/l
ANA titer	0.354	0.1	0.221	0.1	–	
Hemoglobin	14.0	0.2	13.7	0.2	–	13.5–17.5 g/dL
Glucate Hemoglobin	5.7	0.1	5.9	0.2	–	< 6%
BSR (blood sedimentation rate)	42.4	0.3	41.9	0.8	0.063	
Mean Platelet Volume	11.2	0.1	11.0	0.2	–	
*Liver and renal function test*						
Glutamate Pyruvate Transaminase	11.2	0.7	9.9	1.0	–	< 40 U/L
y-Glutamate Transferase	15.8	1.1	12.9	1.7	–	5–50 U/L
Fol_vo	6.7	3.3	20.5	5.1	0.041	4.5–45.3 nmol/L
Ferritin	86.8	8.1	75.1	12.6	–	18–360 µg/mL
Transferrin	3.1	0.1	2.9	0.1	–	2.0–3.6 g/L
Creatinine	0.9	0.1	0.8	0.1	–	0.7–1.2 mg/dL
*Inflammatory mediators*						
C3	114.2	2.3	119.5	3.6	–	75–161 mg/dl
C4	21.5	0.7	25.0	1.0	0.011	16–47 mg/dl
*Microelements in serum*						
Na^+^	139.6	0.2	140.1	0.3	–	135–145 mmol/L
K^+^	4.4	0.1	4.1	0.1	0.054	3.5–5 mmol/L
Cl^−^	103.6	0.9	106.5	1.3	0.092	95–105 mmol/L
Ca^2+^	2.392	0.011	2.394	0.007	0.019	8.5–10.5 mmol/L
Fe^2+^	105.3	3.9	97.4	6.1	–	60–170 µg/dL
Zn^+^	78.3	1.9	73.1	2.9	–	75–110 µg/dL
Vitamin B12	366.7	18.6	424.6	28.6	–	200–900 pg/mL
*Microelements in saliva*						
Zn^+^	4.7	0.4	5.4	0.6	–	
Na^+^	9.7	0.6	11.7	1.0	–	
K^+^	22.7	0.6	18.7	1.0	0.001	
Ca^2+^	1.4	0.1	1.2	0.1	–	

*p*-values after one-way ANCOVA controlled by age

A binary logistic regression was performed to evaluate whether blood biomarkers could predict the presence of taste disorder. Included factors were: age, gender, C4, K, Ca, and transferrin. The model explained 71.6% of the variance and correctly classified 89.9% of the cases (Table 3) and high discrimination AUC = 0.95. Specifically, the model correctly classified 92.0% of controls (92 out of 100) and 84.0% of patients (42 out of 50).

**Fig. 1:**
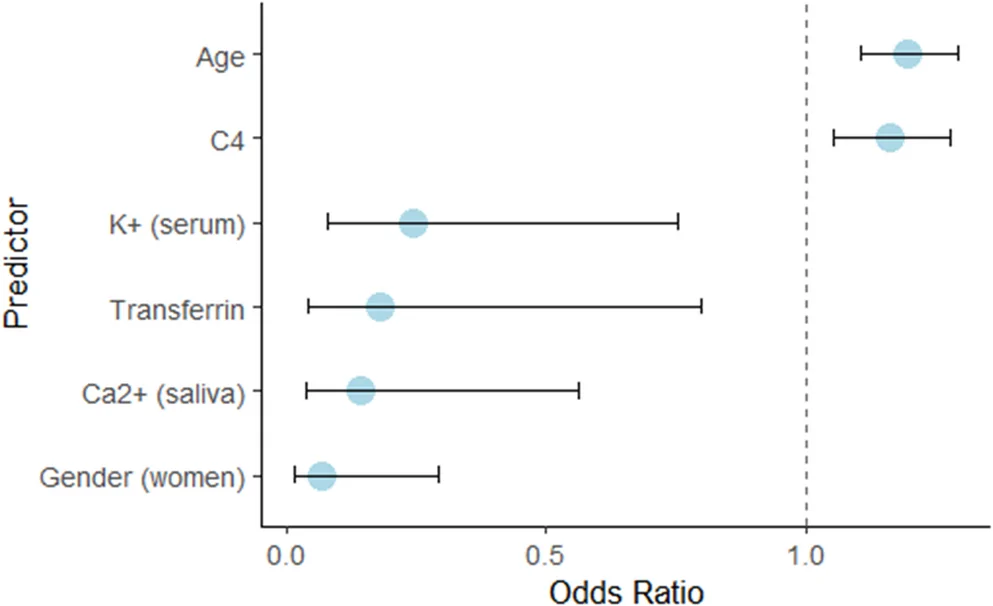
Odds ratios with 95% confidence intervals from the logistic regression model predicting taste disfunction. Variables with OR > 1 are associated with higher odds of the outcome while OR < 1 indicates reduced odds. The dashed horizontal line at OR = 1 represents the point of no effect. The plot is displayed on a logarithmic scale to accommodate the range of OR values

Odds ratios with 95% confidence intervals from the logistic regression model predicting taste disfunction are shown in Fig. 1. Particularly, higher C4 levels were associated with significantly higher odds of having a taste disorder (*p* = 0.004; 95% CI: 0.032–0.273) while higher K in serum, salivary Ca levels, and transferrin were associated with reduced odds of having a taste disorder (*p* ≤ 0.023; 95% CI: −4.289 to − 0.384). Results also showed that increasing age was associated with higher odds of having a taste problem and that males were less likely than females to have taste problems (*p* < 0.001; 95% CI: −5.302 to − 1.292). VIF values for collinearity were low (1.02–1.19).

**Table 3:** Factors that predicted taste dysfunction based on binary logistic regression model

	Factor	β	±SE	Wald	*p*	Exp(B)
Nagelkerke *R*^2^ = 0.730 Correct: 89.9	Age	0.17	0.04	22.11	< 0.001	1.196
	Gender (male)	2.73	0.76	12.90	< 0.001	0.066
	C4	0.15	0.05	8.25	0.004	1.161
	K^+^	-1.42	0.58	6.08	0.014	0.242
	Ca^2+^	-1.97	0.71	7.68	0.006	0.139
	Transferrin	-1.71	0.76	5.15	0.023	0.180

*β* regression coefficient, *SE* standard error

## Discussion

In this study, levels of blood biomarkers were investigated and their concentration were compared to people with idiopathic taste disorders and controls. Among the analyzed parameters, patients with idiopathic taste disorder had higher levels of the complement C4 and Ca in blood serum while concentration of K^+^ in saliva was higher in controls.

C4 is a component of the complement cascade, the immune system pathway which regulates early inflammation and attack on pathogens. Inflammation has increasingly been acknowledged as a significant factor contributing to taste dysfunction. A recent systematic review and meta-analysis revealed that individuals suffering from inflammatory conditions or neurodegenerative diseases such as SARS-CoV-2 infection, chronic rhinosinusitis, chemotherapy, and Alzheimer’s disease are more than three times as likely to experience dysgeusia compared to healthy controls [29]. Even though the patients in our cohort exhibited idiopathic dysgeusia, we noted significantly higher levels of complement C4 in the taste-impaired group in comparison to healthy participants. As recently reported for epithelial cells This observation suggests that activation of the immune system might play a role in idiopathic cases through complement-mediated involvement, and its possible impact on the integrity of taste buds or systemic inflammatory signaling. Collectively, these findings reinforce the idea of inflammation as a common mechanism that underlies dysgeusia, even in the absence of a clearly defined primary cause.

While other studies reported a difference in Zn^2+^, detectable in saliva but not in serum, here Zn^2+^ was not different between groups. Takeda and colleagues demonstrated that zinc deficiency is a predominant factor underlying hypogeusia, even when serum zinc concentrations are within normal ranges [30, 31]. This was supported by their finding that the ACE ratio—defined as the ratio of apo-ACE (zinc-free, inactive form) to holo-ACE (zinc-bound, active form) activities in the serum—was significantly elevated in patients with taste impairment despite normal serum zinc levels. These results suggest that functional zinc status, rather than total serum zinc concentration, may be more closely associated with taste function, an aspect that should be considered when interpreting our findings.

In the taste bud, type I cells exercise a glial-like supportive function by regulating the extracellular K⁺ concentration and maintaining ionic homeostasis [32]. During taste transduction, K⁺ is released into the extracellular space, and type I cells contribute to its clearance and redistribution, thereby restoring ionic homeostasis and preserving the excitability of type II and type III cells. Renal Outer Medullary K^+^ (ROMK) channels, which are selectively expressed in type I cells but absent in type II and III cells, are the primary actors for influx regulation of K^+^ in the cell (ATP controlled) [33]. Potassium, generally more concentrated inside the cytoplasmatic compartment, is a key element for signal transduction and cell functionality. With the low concentration of salivary K^+^ in patients, one can speculate that the concentration might be higher inside the taste bud microenvironment, hyperpolarizing the cell and making it less prone to communications with the downstream nervous fibers. This hypothesis, albeit intriguing, requires confirmation in future studies combining clinical cohorts with functional and molecular analyses, including direct assessment of ionic homeostasis and inflammatory pathway activity.

The binary logistic regression analysis revealed multiple blood biomarkers and demographic variables that significantly predicted the occurrence of a taste disorder. In particular, elevated levels of complement C4, older age, and female sex were correlated with a higher likelihood of experiencing a taste disorder, while higher K^+^, salivary Ca^2+^, and transferrin levels were also associated with a reduced risk. These correlations imply that both immune system activation and imbalances in electrolytes may contribute to the underlying mechanisms of taste dysfunction. Using the same taste test, research on taste function in disorders that alter the concentration of metabolites, such as hypothyroidism, diabetes, and renal dysfunction, showed a correlated decrease in chemosensation, particularly in sweet and bitter perception [34–36]. Consequently, impaired taste perception can reduce dietary enjoyment and alter food choices, often leading to insufficient intake of key nutrients and electrolytes. Such changes in eating behavior can further disrupt metabolic regulation, influencing hydration status, micronutrient balance, and overall energy homeostasis.

Additionally, advancing age heightened the probability of having a taste disorder, consistent with findings in literature that age-related alterations in taste bud regeneration and neural processing lead to diminished taste function [37, 38]. Female sex was also associated with increased odds of taste dysfunction in our cohort, in line with previous reports describing a higher prevalence of taste disorders among women [39].

Given the heterogeneity of the patient cohort, these findings should be interpreted with caution. Despite clinical ENT evaluation, the absence of standardized endoscopic, reflux-related, and full olfactory threshold/discrimination testing may have allowed inclusion of patients with subclinical secondary causes of taste dysfunction. The variability in clinical history within the group limits the ability to draw definitive conclusions or establish a clear-cut diagnostic classification for idiopathic dysgeusia. Results should be interpreted as association with dysgeusia after adjustment for age. Instead, the results should be regarded as preliminary evidence that highlights potential associations at the molecular level, but requires confirmation in larger, more homogeneous samples.

## Conclusion

These findings highlight a multifactorial origin for taste disorders within our idiopathic cohort, where inflammatory processes may interact with metabolic, electrolyte, and demographic factors to influence individual taste perception. These data reinforce the importance of recognizing dysgeusia in ENT practice as a potential marker of systemic disease.

## Data Availability

The data supporting the results of this study are available from the corresponding author upon reasonable request.
